# Germination biology of *Hibiscus tridactylites* in Australia and the implications for weed management

**DOI:** 10.1038/srep26006

**Published:** 2016-05-13

**Authors:** Bhagirath Singh Chauhan

**Affiliations:** 1 The Centre for Plant Science, Queensland Alliance for Agriculture and Food Innovation (QAAFI), The University of Queensland, Toowoomba, Queensland 4350, Australia

## Abstract

*Hibiscus tridactylites* is a problematic broadleaf weed in many crops in Australia; however, very limited information is available on seed germination biology of Australian populations. Experiments were conducted to evaluate the effect of environmental factors on germination and emergence of *H. tridactylites*. Germination was stimulated by seed scarification, suggesting the inhibition of germination in this species is mainly due to the hard seed coat. Germination was not affected by light conditions, suggesting that seeds of this species are not photoblastic. Germination was higher at alternating day/night temperatures of 30/20 °C (74%) and 35/25 °C (69%) than at 25/15 °C (63%). Moderate salinity and water stress did not inhibit germination of *H. tridactylites*. Seedling emergence of *H. tridactylites* was highest (57%) for the seeds buried at a 2 cm depth in the soil; 18% of seedlings emerged from seeds buried at 8 cm but no seedlings emerged below this depth. Soil inversion by tillage to bury weed seeds below their maximum depth of emergence could serve an important tool for managing *H. tridactylites*.

*Hibiscus tridactylites* (known as narrow leaf bladder ketmia in Australia), previously known as *H. trionum* var. *trionum*, is a member of the Malvaceae family^1^. The Malvaceae family is comprised by 80% of the *Hibiscus* genus, making it the largest genus in the family^2^. A recent taxonomic study suggested that *H. tridactylites* is indigenous to Australia^1^. In Australia, it is a common and troublesome weed in maize, sorghum, cotton, and pastures. *H. tridactylites* is closely related to cotton plants in terms of phenological and physiological traits, which makes it very difficult to control in most post-emergent situations^3^. In addition, a number of seedling flushes throughout the season also makes this weed difficult to control. In a previous study, full season weed competition with *H. trionum* (as this name was used in previous studies) reduced soybean yield by up to 75%^4^. In the same study, yield reduction was generally the highest where the weed infestation occurred through the reproductive growth stages. *H. tridactylites* has the following biological traits which contribute to its success as a cropping weed: prolific seed producer (up to 16,000 seeds per plant); high dormancy and long persistence life in the soil; and rapid and competitive plant growth^5^,^6^. In previous studies from elsewhere, about 40% of *H. trionum* seeds survived after 2 years of burial and 25% after 7 years of burial^7^,^8^. Its seed persistence was reported to increase with burial depth from 2 to 10 cm^8^.

Seed germination and seedling emergence of a weed species are affected by several environmental factors. Some of these factors are light, temperature, salt stress, water stress, and seed burial depth in the soil^9^,^10^. Despite the problem of *H. tridactylites* in Australian cropping systems, very limited information is available on its germination and emergence. Better understanding of the factors affecting germination and emergence could facilitate the development of effective cultural management practices for this weed through either suppressing germination or encouraging germination at periods when seedlings can be readily managed^9^. Variable seed dormancy and germination are important survival tactics of weeds. Seeds of some species exhibit a very high level of dormancy^10^,^11^,^12^, while seeds of other species never experience dormancy^13^,^14^. Among different environmental factors, light is an important ecological determinate for germination^9^. No germination in the absence of light means that many seeds will not germinate when buried deep in the soil. Similarly, weed seeds distributed at different soil depths experience different soil temperatures and moisture levels, and their germination is likely to be variable.

Some information about the germination requirements of *H. tridactylites* is available for populations in Greece^3^, but little is available for Australian populations. Although the information on the Greece population can be useful, the germination requirements of *H. tridactylites* in Australia may be different from those of *H. trionum* in Greece.

A study was conducted to determine the effects of scarification, temperature and light, salt and osmotic stress, and burial depth on seed germination and seedling emergence of *H. tridactylites*. It was hypothesized that: a) the germination percentage of *H. tridactylites* seeds will increase with scarification, b) the germination percentage of *H. tridactylites* seeds will decrease linearly with increasing salt and water stress, and c) the percent emergence of *H. tridactylites* seedlings will decrease linearly with increasing seed burial depth in soil.

## Results and Discussion

### Seed scarification

**Figure 1:**
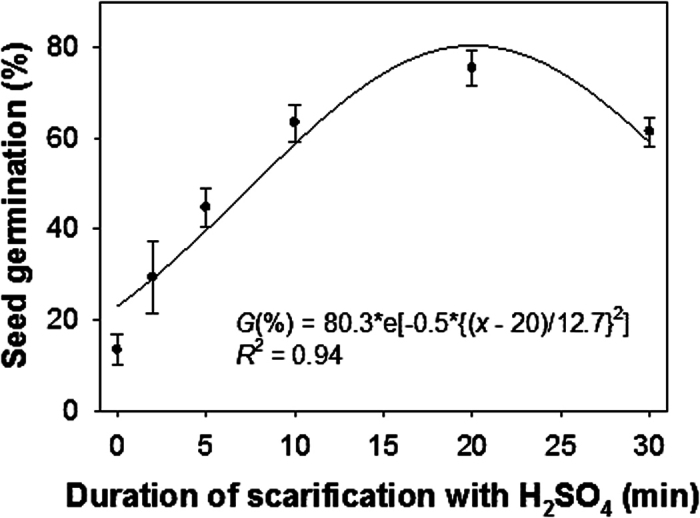
Effect of duration of scarification with sulfuric acid (H_2_SO_4_) on seed germination of *Hibiscus tridactylites* after 14 days of incubation at 30/20 °C alternating day/night temperature. The line represents a three-parameter Gaussian model fitted to the data. Maximum germination was achieved when seeds were scarified with H_2_SO_4_ for 20 minutes.

As hypothesized, chemical scarification with sulfuric acid (H_2_SO_4_) released the seeds from dormancy and stimulated germination. Seed germination increased with the time of H_2_SO_4_ scarification up to 20 minutes and decreased after that (Fig. 1). Only 13% seeds germinated without scarification. The fitted model suggested 20 minutes of scarification with H_2_SO_4_ was required to achieve maximum germination (80%). Therefore, in the subsequent experiments, seeds were scarified with H_2_SO_4_ for 20 minutes.

The previous study in Greece suggested that the optimum acid soaking time to soften the hard *H. trionum* seeds without reducing germination was 30 minutes; however, the authors did not show the data^3^. The seeds of *H. tridactylites* released from dormancy by chemical scarification, suggesting that this species has hard-seeded/physical dormancy. Some other weeds that can break coat-imposed dormancy by scarification are *Malva parviflora* L., *Mimosa invisa* Mart. ex Colla, *Corchorus olitorius* L., and *Melochia concatenata* L^12^,^15^,^16^.

In a previous study in Queensland, Australia, 38% of *H. trionum* seeds remained viable at 0–2 cm depth after 2 years^17^. In another study in Turkey, seeds of *H. trionum* persisted in the soil longer with 23% seeds remaining viable after 7 years^7^. The results of this study suggest that germination of *H. tridactylites* seeds will remain very low in the field unless scarified. Under natural environments, extreme changes in temperature and moisture conditions, soil acids, fire (e.g., window burning practiced in Australia), damage by predators (insects, rodents, microorganisms, etc.), and passage through the digestive system of animals help in seed scarification^9^,^18^. No-till farming systems leave most of the weed seeds on the soil surface after seed rain and crop planting, where they may experience fluctuating temperature and moisture regimes. Viability is usually lost more rapidly for seeds present on the soil surface through germination and mortality than when buried in the soil^19^. In addition, weed seeds are most vulnerable to seed predators when present on the soil surface^9^,^20^,^21^. These observations suggest that *H. tridactylites* seeds can persist for a long time when buried in the soil.

### Temperature and light

Germination of scarified seeds of *H. tridactylites* was not affected (P = 0.7) by light conditions (data not shown), though it was affected (P = 0.002) by the tested temperatures. Germination percentages were greater at 30/20 °C (74%) and 35/25 °C (69%) than at 25/15 °C (63%).

Similar germination responses to light and darkness in *H. tridactylites* indicate that seeds of this species are not photoblastic, and therefore, these seeds may germinate even when buried in the soil or after canopy closure in a crop. These results also suggest that farming practices involving no-till or mulch systems will have no influence in terms of light exposure affecting germination of *H. tridactylites*^16^. Previously, light- independent germination has been reported in several species (e.g., *C. olitorius* and *M. invisa*) who also have a hard seed coat^15^,^16^.

Temperature affects seed germination and governs seasonality and range expansions^16^. Seeds of *H. tridactylites* germinated over the tested range of temperatures, suggesting that seeds of this species can germinate throughout the spring, summer, and autumn seasons in Queensland. These results are consistent with the suggestions provided in a previous study that this species has successive seedling flushes after rainfall or irrigation events throughout spring, summer, and autumn^1^. The previous study using the Greece population also reported highest germination (60%) at 30/20 °C followed by 20/10 °C with 40% germination^3^. These comparisons suggest that both populations may have similar temperature requirements for optimal germination.

### Salt stress

**Figure 2:**
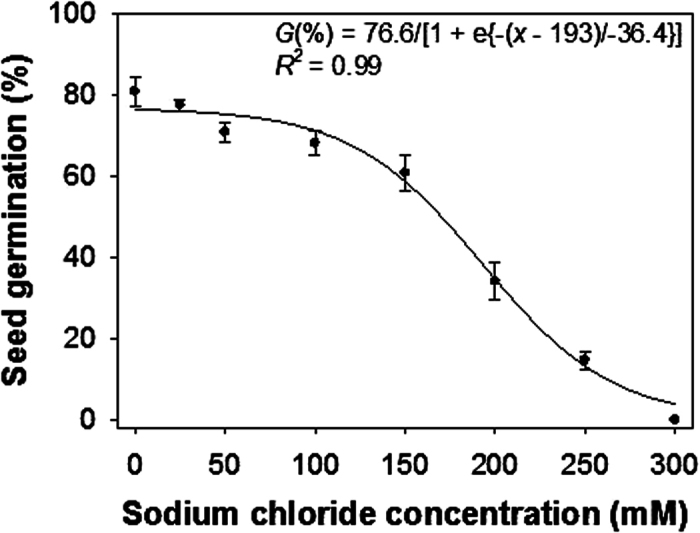
Effect of sodium chloride concentration on germination of scarified seeds of *Hibiscus tridactylites* after 14 d of incubation in light/dark at 30/20 °C alternating day/night temperature. The line represents a three-parameter sigmoid model fitted to the data. The sodium chloride concentration required for 50% inhibition of the maximum germination was 193 mM.

In contrast to the proposed hypothesis, a sigmoid response (rather than a linear response) was observed in the germination of *H. tridactylites* seeds in response to salt concentrations. Maximum germination (77%) was obtained in no-salt conditions (Fig. 2). Germination was greater than 60% up to the concentration of 150 mM sodium chloride (NaCl), 15% germination occurred at 250 mM NaCl, although germination was completely inhibited at 300 mM NaCl. The concentration required for 50% inhibition of maximum germination was 193 mM NaCl.

These results suggest that *H. tridactylites* seeds can germinate in high saline conditions, which could be a key feature of this species enabling it to colonize saline areas. Salinity is an important abiotic stress to crop production worldwide, including Australia. Soils with more than 100 mM NaCl are considered to have high salt contents. In Queensland alone, the area of saline land was 107,000 ha in 2002^22^. Crop production may be affected by soil salinity as well as weed competition. Similar to *H. tridactylites*, seeds of *M. invisa* (another hard-seeded weed species) germinated at very high salt concentrations^15^.

### Water stress

**Figure 3:**
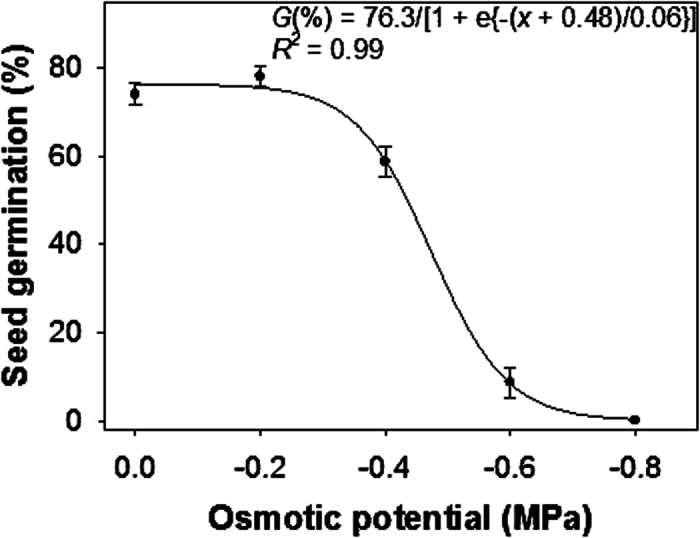
Effect of osmotic potential on germination of scarified seeds of *Hibiscus tridactylites* after 14 d of incubation in light/dark at 30/20 °C alternating day/night temperature. The line represents a three-parameter sigmoid model fitted to the data. The osmotic potential required for 50% inhibition of the maximum germination was −0.48 MPa.

A sigmoid response was observed in the germination of *H. tridactylites* seeds with decreased in osmotic potential from 0 to −0.8 MPa (Fig. 3). However, it was hypothesized that a linear decline in percent germination would be observed in response to increasing water stress. Germination was greater than 74% at osmotic potentials ranging from 0 to −0.2 MPa; 9% of seeds germinated at −0.6 MPa though germination was completely inhibited at −0.8 MPa. The fitted model suggested an osmotic potential of −0.48 MPa to inhibit 50% of maximum germination.

The results of this experiment suggest that *H. tridactylites* seeds can germinate under moderate water stress conditions, which can occur temporarily between the rainfall events at the start of the summer seasons in the northern region of Australia. The ability to germinate under moderate water stress conditions could enable *H. tridactylites* to gain an advantage due to earlier seedling emergence. Similar results were reported in the previous study from Greece and the authors suggested that *H. trionum* germination and establishment would not be halted in poorly drained or mostly dry soil conditions^3^.

### Seed burial depth

**Figure 4:**
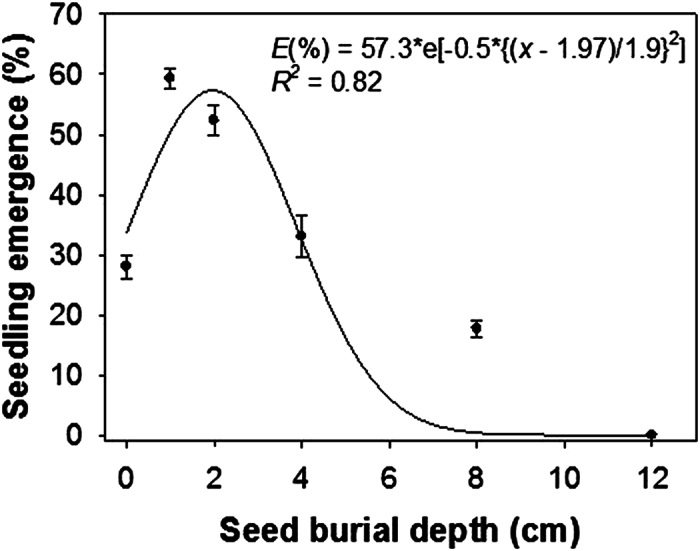
Effect of burial depth on seedling emergence of scarified seeds of *Hibiscus tridactylites.* The line represents a three-parameter Gaussian model fitted to the data. Maximum emergence was achieved at the burial depth of 2.0 cm.

Seedling emergence of *H. tridactylites* was greatly influenced by seed burial depth. The proposed hypothesis was that the percent emergence of *H. tridactylites* seedlings would decrease linearly with increasing seed burial depth in soil. However, this response was not observed. Only 28% of seeds produced seedlings when planted on the soil surface (Fig. 4). Seedling emergence increased with shallow burial at depths of 1 and 2 cm but declined progressively as depth increased. The fitted model predicted a maximum of 57% emergence at a burial depth of 2 cm. Up to 18% of seedlings emerged beyond that depth. Similar results were reported for the Greece population in the previous study^3^. In that study, seedling emergence of *H. trionum* was higher for seeds buried at 2-cm than those placed on the soil surface (54 vs 38%germination).

Seedling emergence on the soil surface was lower than the emergence from the seeds buried at 1 and 2 cm depths. As light is not inhibitory for seed germination in *H. tridactylites*, reduced seedling emergence on the soil surface was probably due to limited soil-seed contact and, consequently, poor seed imbibitions^23^. Low or no emergence from deeply buried seeds could be due to fatal or no germination. Previous studies also suggested limited energy reserves to support hypocotyl elongation from deeper depths^9^,^24^.

The results of this experiment suggest that deep tillage operations, that bury the weed seeds below 8 cm depth, would suppress emergence of *H. tridactylites* seedlings. Subsequent tillage operations, however, should be shallow to avoid the possibility of bringing back the seeds on the soil surface.

## Conclusions

Scarification stimulated germination of *H. tridactylites*, indicating that the hard seed coat is the primary reason for the inhibition of germination. In natural conditions, scarification usually occurs more in seeds present on or near the soil surface than in deeply buried seeds. These observations indicate that the seeds of *H. tridactylites* can persist for a long time when buried deep in the soil. Light did not influence seed germination, suggesting that *H. tridactylites* seeds are not photoblastic. It also indicates that no-till systems will not make any difference in term of light exposure. Seed germination of *H. tridactylites* was moderately tolerant to salt and water stress. Seedling emergence was optimal at shallow burial depths. In case seeds are concentrated in the top soil layer, a deep tillage operation that buries seeds below 8 cm depth would be appropriate. Conclusions drawn from the results of this study should be limited to the weed population sampled because weed populations often vary in their germination requirements. Seed-germination behavior may also differ among seeds produced in different seasons and years. Therefore, conclusions are most relevant to the northern Australian weed population tested.

## Material and Methods

### Seed collection

Seeds of *H. tridactylites* (annual species) were harvested from mature plants grown in fallow fields in Dalby (27.8°S and 151.13°E), Queensland, Australia, in November 2014. The region receives an annual rainfall of around 600 mm. Seeds were collected from approximately 50 plants. The seeds were not enough to conduct several experiments and therefore, these seeds were planted in a field at the research farm of the University of Queensland, Gatton, Queensland. Seeds from these plants were collected in May 2015 for use in the study. Seeds were cleaned, placed in plastic containers, and stored in a laboratory until used in the experiments. Experiments were conducted from May to October 2015.

### Germination test

Seed germination of *H. tridactylites* was evaluated by placing 25 seeds in a 9-cm-diameter Petri dish containing two layers of filter papers. These filter papers were moistened with 5 ml of distilled water or a treatment solution. Seeds used in the experiments were scarified with concentrated sulfuric acid (H_2_SO_4_) for 20 minutes, unless otherwise specified. Petri dishes were placed in transparent plastic bags (to reduce moisture loss from the dishes) and incubated at fluctuating day/night temperatures of 30/20 °C in light/dark conditions. The photoperiod in the incubator was set at 12 h to coincide with the higher temperature interval. Germination was evaluated 14 days after the start of the experiment, with the criterion for germination being visible protrusion of the radical.

### Scarification

An experiment was conducted to investigate whether the seeds of *H. tridactylites* could be released from dormancy by seed scarification. The experiment also aimed to test how long scarification time is needed so that the information can be used for the other experiments of the study. Seeds were scarified with concentrated (98%) H_2_SO_4_ at different time intervals (2, 5, 10, 20, and 30 minutes). The seeds were washed for 5 minutes in running tap water before placing them in dishes. There was also a control treatment in which seeds were not scarified (i.e., 0 minutes with H_2_SO_4_).

### Temperature and light

To evaluate the effect of temperature and light on germination, the chemically scarified seeds of *H. tridactylites* were incubated at three alternating day /night temperatures (25/15, 30/20, and 35/25 °C) in two light regimes [complete darkness (24 h) and light /dark (12 h/12 h)]. These temperature regimes were chosen to reflect the temperature variation during the spring to summer seasons in Queensland, Australia. In the complete darkness treatment, the dishes were wrapped in three layers of aluminum foil (to ensure no light penetration).

### Salt stress

To determine the effect of salinity on germination, the chemically scarified seeds of *H. tridactylites* were incubated in sodium chloride (NaCl) solutions of 25, 50, 100, 150, 200, 250, and 300 mM. There was also a control treatment (0 mM), where distilled water was added.

### Water stress

To determine the effect of water stress (or osmotic stress) on germination, scarified seeds of *H. tridactylites* were incubated with osmotic potential of −0.2, −0.4, −0.6, −0.8, and −1.0 MPa. These solutions were prepared by dissolving polyethylene glycol 8000 in distilled water, as described earlier^25^. There was also a control treatment (0 MPa), in which distilled water was added.

### Seed burial depth

The effect of seed burial depth on the seedling emergence of *H. tridactylites* was studied in a growth chamber (set at 30/20 °C) by placing 50 chemically scarified seeds on or in soil within 15-cm diameter plastic pots. The seeds were placed on the soil surface and covered with the same soil to achieve soil burial depths of 0, 1, 2, 4, 8, and 12 cm. The soil used in this experiment was collected from a nearby field and it was red clay with a pH of 5.5. There was no background seed bank of *H. tridactylites* in the soil. The experiment was initially irrigated with an overhead mist sprinkler and later subirrigated. Seedling emergence of *H. tridactylites* was counted when the cotyledon was easily visible. The experiment was carried out until 35 d after seed burial.

### Statistical analyses

All the experiments were conducted in a randomized complete-block design with three replications. All the experiments were repeated over time (and, therefore a total of six replications) and the second run was started within a month of the termination of the first run. The data variance was visually inspected by plotting the residuals to confirm homogeneity of variance before statistical analysis. ANOVA indicated no difference between the experiments conducted at the different times and therefore, the data from the two experimental runs were combined for analysis.

The data obtained from the temperature and light experiments were separated using the least significant difference (LSD) at 5% (Gen Stat 16^th^ Edition, VSN International Ltd, United Kingdom), while regression analysis was used for other experiments (SigmaPlot 10.0, Systat Software, Inc; Pint Richmond, CA).

A three-parameter sigmoid model was fitted to the germination values resulting from salt and water stress experiments. The model was


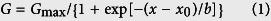


where, *G* the total germination (%) at NaCl or osmotic potential *x*; *G*_max_ is the maximum germination; *x*_*0*_ is the NaCl or osmotic potential required for 50% inhibition of maximum germination; and *b* is the slope of the curve.

A three-parameter Gaussian model was fitted to the germination values resulting from the scarification experiment and seedling emergence values resulting from the seed burial experiment. The model was:


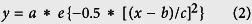


The graph of a Gaussian is a “bell curve” shape. In the model, *a* is the height of the curve’s peak (i.e., maximum germination or emergence); *b* is the position of the center of the peak (i.e.; the duration of scarification required to achieve maximum germination or the depth of seed burial to achieve maximum seedling emergence); and *c* is the width of the “bell”.

## Additional Information

**How to cite this article**: Chauhan, B. S. Germination biology of *Hibiscus tridactylites* in Australia and the implications for weed management. *Sci. Rep.* **6**, 26006; doi: 10.1038/srep26006 (2016).
